# Evaluation of the anti-ischemic effects of D-ribose during dobutamine stress echocardiography: a pilot study

**DOI:** 10.1186/1476-7120-7-5

**Published:** 2009-02-07

**Authors:** Stephen G Sawada, Stephen Lewis, Roxanne Kovacs, Samer Khouri, Irmina Gradus-Pizlo, John A St Cyr, Harvey Feigenbaum

**Affiliations:** 1 Krannert Institute of Cardiology, The Department of Medicine of the Indiana University School of Medicine, The Richard Roudebush Veterans Affairs Medical Center, Indianapolis, IN, USA; 2 Wishard Memorial Hospital, Indianapolis, IN, USA; 3 Bioenergy, Inc. Research Division, Minneapolis, MN, USA

## Abstract

D-Ribose, a pentose sugar, has shown to improve myocardial high-energy phosphate stores depleted by ischemia. This study investigated the ability of D-Ribose with low dose dobutamine to improve the contractile response of viable myocardium to dobutamine and to assess the efficacy of D-ribose in reducing stress-induced ischemia. Twenty-six patients with ischemic cardiomyopathy completed a two-day, randomized, double blind crossover trial comparing the effects of D-Ribose and placebo on regional wall motion. On the first study day, either D-Ribose or placebo was infused for 4.5 hours. Low (5 and 10 μ/kg/min) and subsequently, high (up to 50 μ/kg/min) dose dobutamine echocardiography was then performed. On the second study day, patients crossed over to the alternative article for a similar 4.5 hours infusion time period and underwent a similar evaluation. The wall motion response during low dose dobutamine was the same with D-Ribose and placebo in 77% of segments (203/263, Kappa = 0.37). In segments with discordant responses, more segments improved with D-Ribose than with placebo (41 vs. 19 segments, p = 0.006). With high dose dobutamine infusion, the wall motion response (ischemia vs. no ischemia) was the same with D-Ribose and placebo in 83% of interpretable segments (301/363, kappa = 0.244). In segments with discordant responses, there were more ischemic segments with placebo compared to D-Ribose (36 vs. 26, p = 0.253). Nineteen patients developed ischemia during the dobutamine and placebo infusion and 13 patients had ischemia during dobutamine and D-ribose infusion (p = 0.109). D-Ribose improved contractile responses to dobutamine in viable myocardium with resting dysfunction but had no significant effect in reducing the frequency of stress-induced wall motion abnormalities.

## Background

The maintenance of cellular integrity and contractility rely on adequate levels of adenosine triphosphate (ATP). Myocardial ischemia alters oxidative metabolism resulting in lower tissue ATP levels along with an associated impairment in contractile function [1,2]. The repletion of ATP stores depends on the availability of the precursor phosphoribosyl-pyrophosphate (PRPP). D-Ribose (DR), a pentose monosaccharide, has been shown to accelerate ATP repletion in the ischemic myocardium by promoting the production of PRPP. In animal studies, DR enhanced the repletion of ATP after global and regional ischemia, resulting in an improvement in contractile function [3-7]. Pliml et al. showed that DR reduced the frequency of stress-induced ST segment depression in patients with stable coronary arterial disease [8]. The effect of DR in ischemic contractile dysfunction in patients is unknown.

The anti-ischemic effects of DR were tested during low and high dose dobutamine infusion in patients with ischemic left ventricular systolic dysfunction. Low dose dobutamine infusion is widely used for the assessment of viability in segments with resting dysfunction due to hibernating or stunned myocardium [9]. The lack of a contractile response with dobutamine in viable segments may be due to the tenuous oxygen supply-demand balance that exists in segments supplied by severely diseased vessels [10,11]. Viable myocardium may fail to contract during low dose dobutamine stimulation if there is an increase in oxygen demand due to existing native vascular ischemia. D-Ribose may improve ATP levels in a setting where inotropic infusion increases oxygen demand. The effect of DR in preventing or reducing stress-induced contractile dysfunction was also assessed during high dose dobutamine infusion.

## Methods

### Patient Selection

Potential subjects for the study were screened by review of echocardiograms performed for clinical purposes at Indiana University Medical Center. Patients with (1) left ventricular dysfunction (ejection fraction < 50%), (2) resting regional wall motion abnormalities, and (3) coronary artery disease were eligible for the study. Coronary artery disease was documented by one or more of the following: coronary angiography, stress testing showing ischemia, or previous myocardial infarction. Only patients with stable symptoms were included. Patients requiring daily or frequent adjustment of vasoactive medications were excluded as well as those who had myocardial infarction or an episode of unstable angina less than 5 days before enrollment. Subjects having diabetes mellitus requiring insulin or oral hypoglycemic medications were also excluded on the basis of studies showing that plasma glucose levels may decline with DR administration [12].

Twenty-eight patients (25 men, 3 women) with a mean age of 56 ± 12 years were enrolled in this study. All patients had reduced left ventricular systolic function (mean ejection fraction 30 ± 8%, range 16 to 49%). Twenty-five patients had a prior history of myocardial infarction. Twenty-one patients had stable angina pectoris and were receiving 1 or more medications, including nitrates, beta-antagonists, and calcium channel antagonists. Coronary artery disease (≥ 50% diameter stenosis of a major coronary artery) was documented by angiography in 25 patients. Of the 24 patients with recent angiograms, 12 had three vessel disease, 10 had two vessel disease, and 2 had single vessel disease.

### Study Protocol

This randomized double-blind placebo-controlled trial was approved by the Indiana University Institutional Review Board and informed consent was obtained from all patients. The study was performed at the Clinical Research Center of Indiana University Hospital. The University Hospital Pharmacy performed the randomization and blinding of each test article. D-Ribose was administered as a 10% solution in water at 180 mg/kg/hr, a dose which has previously shown in humans to be without adverse effects [13].

On Day 1, patients underwent an initial physical examination and a baseline echocardiogram. Peripheral intravenous catheters were placed and the randomized test article (DR or placebo) was infused along with a separate infusion of D5W (1.8 ml/kg/hr) at 100 ml/hr as a maintenance fluid. Four and one half hours following commencement of the test article infusion, a second echocardiogram was performed. After this imaging study, dobutamine infusion was initiated. Echocardiograms were acquired at low doses (5, 10 μg/kg/min) and at a peak dose of up to 50 μ/kg/min. Upon completion of all imaging, the infusion was terminated. Patients were observed overnight in the Research Center and on Day 2 were crossed over to the alternate test article. The procedures performed on Day 2 were the same as that of Day 1. Patients were discharged to home after several hours of observation following completion of the procedures on Day 2.

### Dobutamine Stress Echocardiography

Dobutamine stress echocardiograms were obtained using parasternal long and short axis and apical two and four chamber views. Dobutamine was initially infused at 5 μg/kg/min, increased to 10 μg/kg/min after 3 min and then by 10 μg/kg/min increments every 3 min. Endpoints for the infusion included: extensive new or worsening wall motion abnormalities; chest pain, ≥ 2 mm ST segment depression or elevation; significant adverse side effects, arrhythmias or hypotension (defined by the supervising physician); ≥ 85% of the age-predicted maximal heart rate, or at the maximal dose of 50 μg/kg/min. Intravenous atropine was used in subjects who did not have substantial increases in heart rate at the 30 μg/kg/min dose. Echocardiographic images were digitally stored at rest, 5 and 10 μg/kg/min, and at peak dose.

Myocardial wall motion and thickening were visually assessed by the consensus of two investigators using a 16 segment model of the left ventricle and a previously described scoring system [14]. Investigators were blinded to the clinical data and test articles (DR or placebo). Each dobutamine study arm was analyzed separately without direct comparisons of Day 1 and Day 2 images. Segments with a baseline score of ≥ 2.0 were considered to have resting dysfunction. Improvement in function by one grade with low dose dobutamine was considered to be significant. Stress-induced ischemia with high dose dobutamine was defined by the development of a new wall motion abnormality or worsening of a pre-existing wall motion abnormality except for segments that were akinetic both at rest and at low dose dobutamine.

### Statistical Analysis

Data for heart rate, blood pressure, regional wall motion scores and ejection fraction are expressed as the mean ± one standard deviation (SD). Resting hemodynamic data obtained with either DR or placebo were assessed with paired t-tests. The changes in these parameters during dobutamine infusion were compared using two-way repeated measured ANOVA. A significant interaction term indicated that the response to dobutamine was different on DR compared to placebo. Agreement in myocardial segmental responses to dobutamine + DR or dobutamine + placebo was evaluated by the Kappa statistic. The significance of differences in the response of individual patients and myocardial segments to dobutamine + DR and dobutamine + placebo was determined by McNemar's test. Categorical variables were analyzed by the Fisher exact test. A p value < 0.05 was considered significant.

## Results

Of the 28 patients enrolled in the study, two failed to complete the two day study protocol. One patient developed chest pain during infusion of the placebo on Day 2. The chest pain was relieved with sublingual nitroglycerin and dobutamine echocardiography was not performed. The following day, coronary angiography demonstrated obstruction of the posterolateral branch of the right coronary artery which was treated with angioplasty. The second patient developed chest pain on the first study day during infusion of dobutamine and placebo. The patient was taken for urgent coronary angiography with angioplasty of a severely stenotic diagonal artery.

### Hemodynamics

In the 26 patients who completed both study days, resting left ventricular ejection fraction was similar between DR and placebo (31 ± 8%, 30 ± 8%, respectively, p = 0.945). The peak dose of dobutamine used was 42 ± 11 ug/kg/min in the placebo group and 42 ± 8 uk/kg/min in the DR patients (p = 0.770). Hemodynamic measurements in both DR and placebo groups are shown in Table 1. There was a trend towards a higher rate pressure product at rest in the placebo group (p = 0.09). The hemodynamic response to both low and high doses of dobutamine was similar in both the DR and placebo groups. Rate pressure product increased significantly from rest to low dose dobutamine infusion (10 ug/kg/min) in both the DR (p = 0.001) and the placebo groups (p = 0.004). The increase in rate pressure product at 10 ug/kg/min of dobutamine was similar between the two test articles (p = 0.330) and the peak (high dose dobutamine) rate pressure product was also similar between placebo and DR (16,060 ± 5050 vs. 15,540 ± 4560, respectively, p = 0.569).

**Table 1:** Hemodynamic Parameters at Rest and During Dobutamine (Dob) Infusion

	HR	SBP	DBP	HR × BP		HR	SBP	DBP	HR × BP
AM	68 ± 11	123 ± 19	74 ± 13	8390 ± 2040	AM	71 ± 13	122 ± 13	77 ± 10	8640 ± 2240

PM	67 ± 13	116 ± 14	71 ± 9	7860 ± 1990	PM	69 ± 14	125 ± 20	72 ± 10	8690 ± 2220

Dob Rest	64 ± 14	116 ± 15	67 ± 11	7500 ± 1900	Dob Rest	65 ± 13	123 ± 18	73 ± 12	8090 ± 2110

Dob 5 μg	66 ± 15	116 ± 15	66 ± 11	7720 ± 2030	Dob 5 μg	68 ± 14	121 ± 19	70 ± 15	8320 ± 2390

Dob 10 μg	74 ± 17	118 ± 15	65 ± 10	8750 ± 2480	Dob 10 μg	75 ± 20	122 ± 20	70 ± 13	9300 ± 3540

Peak	120 ± 18	121 ± 19	62 ± 11	14570 ± 3070	Peak	119 ± 24	130 ± 24	67 ± 14	15460 ± 4780

### Regional Function Assessment at Low Dose Dobutamine

The results of DR +low dose dobutamine and placebo + low dose dobutamine on a per patient basis are represented in Table 2. Improvement in at least one myocardial segment was required for a patient to be considered as having improvement in regional wall motion. The overall agreement between DR and placebo was 65% (17/26), kappa = .22. In the 9 patients with discordant findings, 6 had improvement with DR + dobutamine vs. 3 with placebo + dobutamine (p = 0.508). The segment by segment correlation between the results of DR + dobutamine and placebo + dobutamine is shown in Table 3. Overall agreement was 77% (203/263), kappa = 0.37. In 60 segments with discordant responses, an improvement in wall motion during low dose dobutamine infusion occurred more frequently with DR than with placebo (41 vs. 19 segments, respectively, p = 0.006).

**Table 2:** Patients With and Without Improvement in Regional Wall Motion during Low Dose Dobutamine Infusion

	Dob + DR	
	Yes	No
Yes	13	3*
Dob + Placebo		
	
No	6*	4

Dob = low dose dobutamine. *p = 0.508 that the frequency of improvement with Dob + DR is similar to Dob + placebo.

**Table 3:** Segments With and Without Improvement of Regional Wall Motion with Low Dose Dobutamine

	Dob + DR	
	Yes	No
Yes	31	19*
Dob + Placebo		
	
No	41*	172

p* = 0.006 (frequency of improvement is the same with dobutamine + DR and dobutamine + placebo)

### Regional Function with High Dose Dobutamine Stress

The results with high dose DR + dobutamine and placebo + dobutamine on a per patient basis are shown in Table 4. The overall agreement between DR and placebo was 62% (16/26), kappa = 0.23. In the 10 patients with discordant findings, 8 patients developed ischemic wall motion abnormalities with placebo compared to 2 with DR (p = 0.109). The segment by segment correlation between dobutamine + DR or dobutamine + placebo is shown in Table 5 with an overall agreement of 83% (301/363), kappa = 0.24. In the 62 myocardial segments with discordant results, there was no significant difference in the number of ischemic segments with placebo vs DR infusion (36 vs 26 segments, respectively, p = 0.253).

**Table 4:** Patients With and Without Stress-Induced Wall Motion Abnormalities

	Dob + DR	
	Yes	No
Yes	11	8*
Dob + Placebo		
	
No	2*	5

*p = 0.109 (frequency of stress-induced wall motion abnormalities with Dob + DR is similar to that for Dob + placebo)

**Table 5:** Segments With and Without Stress-Induced Wall Motion Abnormalities

	Dob + DR	
	Yes	No
Yes	16	36*
Dob + Placebo		
	
No	26*	285

p* = 0.253 (frequency of stress-induced wall motion abnormalities with Dob + DR is similar to that for Dob + placebo)

## Discussion

This pilot study revealed that intravenous DR improved the contractile response of segments with resting dysfunction to low dose dobutamine infusion. DR did not significantly reduce the effects of stress-induced ischemia.

### Efficacy of D-ribose with Low Dose Dobutamine

Myocardial ischemia depletes ATP stores. Adenosine triphosphate stores normally regenerate slowly through a salvage pathway that recycles nucleotide precursors [15]. D-ribose improves repletion of ATP stores by increasing precursors of ATP. In animal models, the improvement of ATP stores has been correlated with improvements in contractile function.

In this study, DR improved the contractile response to dobutmaine in myocardial segments with resting dysfunction. Viable segments with resting dysfunction are presumed to have repetitive stunning or a progressive reduction in resting perfusion characterized as hibernation. Adenosine triphosphate stores are reduced in hibernating myocardium [16,17]. Hibernating myocardial segments are typically supplied by severely stenotic arteries, which result in absent or nearly an absent perfusion reserve. Therefore, oxygen supply-demand balance is tenuous. The administration of low dose dobutamine may increase myocardial oxygen demand, which can further deplete ATP stores, preventing or limiting any improvement in regional contractility with inotropic stimulation [18]. We found a small but significant increase in heart rate – blood pressure product during low dose (10 μg/kg/min) dobutamine infusion, reflecting an increase in myocardial oxygen demand. In a previous study, low dose dobutamine has been shown to worsen the reduction in myocardial ATP levels in the presence of reduced resting perfusion [19].

### Clinical Utility of D-Ribose and Low Dose Dobutamine Infusion

Our findings suggest potential uses of DR both as a diagnostic and a therapeutic agent. Low dose dobutamine infusion is used as a diagnostic technique for the detection of viable myocardium in patients who are considered candidates for coronary revascularization. This technique may have reduced sensitivity in patients who have reduced resting myocardial perfusion and hibernation [10,14]. Underestimation of the presence or extent of viable myocardium may lead to a decision not to perform revascularization or in a delay in revascularization. Studies have shown that a delay in revascularization of hibernating myocardium may lead to myocardial infarction and cardiac death [20,21].

Low dose dobutamine infusion also remains as a therapeutic modality for short-term inotropic support for congestive heart failure patients with low cardiac output; however, the use of inotropics can be potentially harmful [22,23]. Induction of arrhythmias or ischemia with inotropic infusions may be the mediators of a poorer clinical outcome. In a swine model of hibernation, Schulz, et al. reported that low dose dobutamine administered for one and a half hours produced a progressive reduction in ATP levels and myocardial necrosis [19]. In rodent studies, ATP stores and the magnitude of improvement in contraction with isoproterenol infusion declines with prolonged administration of the catecholamine. D-Ribose been shown to improve ATP stores and contractile function in rats subjected to prolonged isoproterenol infusions [24]. D-Ribose has also been shown to reduce the frequency of isoproterenol induced myocardial necrosis [25]. D-ribose, by improving the contractile response to dobutamine, may also permit the use of lower doses of dobutamine to achieve the desired hemodynamic improvement in stroke volume.

### Efficacy of D-Ribose in Stress-Induced Ischemia

D-Ribose did not significantly reduce the frequency or extent of myocardial ischemia during high dose dobutamine infusion. Heart rates and rate-pressure products at peak stress were comparable between the infusions of placebo and DR, suggesting that the level of stress was similar. These preliminary findings suggest that DR may not offer an adequate protection during acute ischemic situations demanding marked increases in oxygen demand. However, in our study, there was a trend towards fewer patients having stress-induced ischemia with DR compared to placebo. Furthermore, in our pilot study a relatively brief (4.5 hour) infusion period of DR was used. Restoration of normal myocardial ATP levels may require at least 24 hours following a moderate ischemic episode [26]. Pliml et al. showed that DR was effective in reducing ischemic ST segmental changes during exercise testing when given orally for 3 days before evaluation [8]. Our results suggest that in patients who may have reduced ATP levels from prolonged or repetitive episodes of ischemia, an extended infusion period of DR may be required to demonstrate efficacy for the prevention of stress-induced ischemia.

## Competing interests

JASC is a consultant for Bioenergy. Inc., holding Bioenergy, Inc. warrants/stock options. All other authors declare that they have no competing interests.

## Authors' contributions

SGS was the principle investigator of the study and was responsible for data analysis, writing/revisions of manuscript, approval of final manuscript. SL was involved in study, data analysis, writing/revisions of manuscript. RK was the coordinator of study and was responsible for the tabulation of data, editorial assistance of manuscript. SK was involved in study, data analysis, writing/revisions of manuscript. IGP was involved in study, analysis of data, writing/revisions of manuscript. JASC assisted in the writing/revisions of manuscript, approval of final manuscript. HF was involved in data analysis, writing/revisions of manuscript, approval of final manuscript.
